# Uropathogenic *Escherichia coli* Releases Extracellular Vesicles That Are Associated with RNA

**DOI:** 10.1371/journal.pone.0160440

**Published:** 2016-08-08

**Authors:** Cherie Blenkiron, Denis Simonov, Anita Muthukaruppan, Peter Tsai, Priscila Dauros, Sasha Green, Jiwon Hong, Cristin G. Print, Simon Swift, Anthony R. Phillips

**Affiliations:** 1 Department of Molecular Medicine and Pathology, University of Auckland, Auckland, New Zealand; 2 Department of Surgery, University of Auckland, Auckland, New Zealand; 3 Department of Obstetrics and Gynaecology, University of Auckland, Auckland, New Zealand; 4 Bioinformatics Institute, University of Auckland, Auckland, New Zealand; 5 School of Biological Sciences, University of Auckland, Auckland, New Zealand; 6 Maurice Wilkins Centre, University of Auckland, Auckland, New Zealand; Centre National de la Recherche Scientifique, Aix-Marseille Université, FRANCE

## Abstract

**Background:**

Bacterium-to-host signalling during infection is a complex process involving proteins, lipids and other diffusible signals that manipulate host cell biology for pathogen survival. Bacteria also release membrane vesicles (MV) that can carry a cargo of effector molecules directly into host cells. Supported by recent publications, we hypothesised that these MVs also associate with RNA, which may be directly involved in the modulation of the host response to infection.

**Methods and Results:**

Using the uropathogenic *Escherichia coli* (UPEC) strain 536, we have isolated MVs and found they carry a range of RNA species. Density gradient centrifugation further fractionated and characterised the MV preparation and confirmed that the isolated RNA was associated with the highest particle and protein containing fractions. Using a new approach, RNA-sequencing of libraries derived from three different ‘size’ RNA populations (<50nt, 50-200nt and 200nt+) isolated from MVs has enabled us to now report the first example of a complete bacterial MV-RNA profile. These data show that MVs carry rRNA, tRNAs, other small RNAs as well as full-length protein coding mRNAs. Confocal microscopy visualised the delivery of lipid labelled MVs into cultured bladder epithelial cells and showed their RNA cargo labelled with 5-EU (5-ethynyl uridine), was transported into the host cell cytoplasm and nucleus. MV RNA uptake by the cells was confirmed by droplet digital RT-PCR of *csrC*. It was estimated that 1% of MV RNA cargo is delivered into cultured cells.

**Conclusions:**

These data add to the growing evidence of pathogenic bacterial MV being associated a wide range of RNAs. It further raises the plausibility for MV-RNA-mediated cross-kingdom communication whereby they influence host cell function during the infection process.

## Introduction

Bacteria communicate to one another using diffusible signals [1] and also to the host during the infection process using well-studied proteins, amino acids and lipids and small molecule signals [2]. This cross-kingdom signaling allows the bacterium to manipulate the host defense mechanisms to promote its survival. Bacterial signals are also known to be carried in cargo-containing membrane vesicles (MV), released by the bacterium [3]. The production of MVs, particularly by Gram negative bacteria, is thought to be important not only in bacterium to bacterium signaling but also in bacterium-host signaling [4]. During infection MVs carry toxins and virulence factors to host cells and shield the infecting bacteria from the host immune response and allow colonization [4]. The vesicle structure also allows the packaging of insoluble material, provides protection from external enzymes and antibodies and provides a direct mechanism for docking with the target host cell. In the case of the Uropathogenic *Escherichia coli* (UPEC) strain 536, the pathogenic bacterium used in this study, the MVs have been reported to contain the protein toxin hemolysin [5]. Other UPEC strains release another toxin, cytotoxic necrotizing factor type 1 (CNF1) in MVs, which targets the Rho GTPase signaling pathway to modulate the acute inflammatory response in host cells [6].

To date the bacterium-to-host signaling focus has been largely on the protein and lipids as the effector molecules, particularly in the study of MV cargos. However it has recently been noted that ‘bacterial silencing suppressors may derive from regulatory RNAs instead of proteins…’ [7]. There has been a growing interest in the role of RNAs as novel intercellular signals between kingdoms such as between the nematode *Caenorhabditis elegans* and *E*. *coli* [8] and between the fungus *Botrytis cinera* and plant *Arabidopsis* [9]. In these two examples, cross-species RNA signals function within the host cell via exploitation of RNA-inhibition (RNAi), either blocking the protein machinery as in the case of the fungal-plant interaction or by using the RNAi pathway to directly inhibit host gene translation.

Recently, three groups have reported that a variety of bacteria release MVs that are associated with RNA [10–12]. These ranged from a marine picoplankton *Prochlorococcus* to non-pathogenic *E*. *coli* to *Vibrio cholerae*, the water borne bacterium that causes Cholera. In parallel timeframes we studied, and now present here, the existence of MV associated RNA population derived from uropathogenic *E*. *coli* strain, 536.

In this study we have focussed on the analysis of the RNA content and uptake of UPEC MVs. We provide the first report confirming that UPEC MVs are associated with RNA as well as using a new method to provide a comprehensive sequence profile of this cargo. We also demonstrate the uptake of MVs and their associated RNA into target human bladder epithelial cells *in vitro*.

## Methods

### Bacterial growth and viability assay

Uropathogenic *Escherichia coli* (UPEC) strain 536 (O6:K15:H31) [13] was grown to exponential phase (Optical Density at 600nm, OD600≈1.5) in 10mL of RPMI 1640 medium (Thermo Fisher Scientific) supplemented with 10μM FeCl_3_ (RF), at 37°C with shaking at 200 r.p.m. and then diluted 1:100 in RF (OD600≈0.015) to be grown to stationary phase for ~16 hours overnight. After overnight incubation (OD600≈2.5) the culture was assessed for viability using the LIVE/DEAD BacLight kit (Thermo Fisher Scientific) using the manufacturers recommended protocol. OD readings were taken and cuvettes with a 1 cm path length.

Bacterial cells were removed by centrifuging twice at 7,000 x*g* for 10 min at 4°C, after which any residual cells were removed from the supernatant by filtration using 0.22μm PES filter (Merck Millipore). Supernatants were concentrated using 100 kDa Vivaflow 200 cassettes (Sartorius AG), which removes proteins/molecules under 100 kDa, and the vesicles pelleted by centrifugation at 75,000 x*g* for 2.5hr at 4°C. Vesicles were resuspended in 20mM HEPES or PBS, filter sterilised using a 0.22μm PES filter syringe and again concentrated using 100 kDa Vivaspin 500 columns (Sartorius AG) and stored at -80°C.

### Density gradient centrifugation

The crude MV preparations were further purified by density gradient centrifugation (DGC). A 6 layer (1.8 ml of 45, 40, 35, 30, 25 and 20%) OptiPrep density gradient medium (Sigma-Aldrich) was set up in 12 mL ultracentrifugation tube (UltraClear 5/8 x 3 ¾ in, Beckman Coulter). The crude preparation adjusted to the 45% medium was loaded and the tube was centrifuged at 100,000 x*g* for 16 hours at 4ᵒC (rotor JS-24 and centrifuge Avanti J-30 I, Beckman Coulter). Resulting fractions were determined by visual inspection and removed from the top and diluted in ~ 50 mL of PBS. Fractions were then concentrated with 100 kDa Vivaspin 20 columns to volumes of ~600–900 μL. Aliquots for estimates of protein and RNA content and particle analysis were taken from these fractions and the remainder was stored at -80ᵒC.

### Protein analysis of MV preparations

Vesicle preparations were quantified for protein content using bicinchoninic acid (BCA) assay (Thermo Fisher Scientific), according to the manufacturer’s instructions and protein profiles assessed with 10μg sample loaded onto NuPAGE Bis-Tris 4–12% gradient gels (Thermo Fisher Scientific) and Coomassie Blue staining.

### Nanosight tracking analysis

MV preparations were analysed using Nanosight NS300 system (Malvern Instruments Ltd.) and data analysed using NTA software version 3.0. Each sample was diluted 100–10000 times, administered at constant flow with a syringe pump at 25°C and recorded in sets of three videos of 30 s with 5 s delay between recordings.

### Transmission electron microscopy (TEM)

Bacterial cultures (1mL) were pelleted by centrifuging at 7,000 x*g* for 10 min at 4°C then fixed in 2.5% (w/v) glutaldehyde in 0.1M phosphate buffer (pH 7.4) for 1hr at room temperature. Fixed pellets were post-fixed for 1hr at room temperature in 1% (w/v) osmium tetroxide in 0.1M phosphate buffer, dehydrated in a graded series of ethanol and propylene oxide, embedded in Agar 100 epoxy resin (Agar Scientific, Stansted, Essex, UK) and polymerised at 60°C for 48 hr. Ultrathin sections of 80nm were cut on a Leica Ultracut UCT ultramicrotome (Leica, Wetzler, Germany) and collected on copper grids (ProSciTech Pty. Ltd., Australia). Sections were stained with uranyl acetate and lead citrate.

For negative staining, MV preparations were adsorbed onto Formvar coated copper grids for 2 min. Excess sample was blotted off with filter paper. The grid was then transferred to a drop of 2% (w/v) aqueous uranyl acetate for 2 min before blotting off the excess and air drying the grids. All grids were viewed in a Tecnai G^2^ Spirit TWIN transmission electron microscope (FEI, Hillsboro, OR, USA) at 120 kV accelerating voltage. Images were captured using a Morada digital camera (SIS, GmBH, Munster, Germany).

### Nucleic acid extraction from membrane vesicles

DNA was isolated from vesicle preparations using a GES method [14]. DNA content was assessed by spectrophotometry (Nanodrop Technologies Inc.) and quantitative PCR using primers for *rrsG* (F- 5’-CGTGTTGTGAAATGTTGGGTTAA; R- 5’- CCGCTGGCAACAAAGGATAA).

For RNA, vesicle preparations were resuspended in up to 1mL of TRIzol (Thermo Fisher Scientific), with 20μg of glycogen (Thermo Fisher Scientific) and 200μL of chloroform. Samples were vortexed for 15 sec, incubated for 10 min at room temperature and centrifuged at 17,000 x*g* for 10 min at 4°C. The aqueous phase was removed and mixed with an equal volume of 100% ethanol, and purified using a Purelink RNA mini column (Thermo Fisher Scientific) according to the manufacturer’s protocol for total RNA. Yields and purity were determined by spectrophotometry and RNA integrity was assessed using a TapeStation (Agilent Technologies) or Agilent Bioanalyser (Agilent Technologies). RNA for sequencing was separated into the small and large RNA fractions (><200bp) with an Ambion miRvana RNA isolation kit (Thermo Fisher Scientific) following the procedure for RNA clean-up.

### RNA-sequencing

MV RNA was isolated from UPEC 536. Libraries were prepared using a TruSeq small RNA library kit (Illumina), retaining the fractions from the gel isolation step that correspond to 15-50bp and 50-200bp. These were run on an Illumina MiSeq sequencing machine (2x50bp reads and 2x250bp reads, respectively), one library per MiSeq lane.

Libraries of longer RNAs were prepared using an Illumina TruSeq stranded mRNA kit, excluding the poly-A selection step and without ribo-zero depletion (no removal of ribosomal RNAs). The ribo-zero step was deliberately excluded in order to provide us with a full understanding of the RNA content of UPEC MVs, including these highly abundant ribosomal RNAs. 60ng of total RNA was used as an input for the fragment, prime and finish step of the protocol. One library was run on each lane of a HiSeq 2500 sequencing machine (2x100bp). RNA-sequencing was performed as a contract service by New Zealand Genomics Ltd.

### Bioinformatics Analyses

Data was linker trimmed using fastq-clipper (http://hannonlab/cshl/edu/fastx_toolkit/) then quality and length filtered (Q<20, length <20) using SolexaQA [15]. Reads were then mapped against the genome for UPEC 536 obtained from Ensembl (Genome assembly GCA_0000113305.1 [16]) using BWA-MEM [17] with standard mapping parameters and annotated in SeqMonk using Refseq and RFAM 9.1 databases. Non-unique mapped reads were sited once in the genome for identification, these mostly mapped to tRNA and rRNA genes. Data are available online from Sequence Read Archive (SRA/GEO) Accession SRP079272.

### Infection of bladder cells with membrane vesicles

5637 bladder carcinoma cells were purchased from the American Type Culture Collection (Manassas, VA, USA) and cultured in RPMI 1640 containing 10% vol/vol foetal bovine serum (MediRay, New Zealand) in humidified air with 5% vol/vol CO_2_ at 37°C. The cells were seeded (100,000 cells/mL) in full growth media and incubated overnight for 15 hr before addition of membrane vesicles and further incubation at 37°C for various timeframes, up to 96 hr. At specified timepoints cells were washed twice with PBS and placed into TRIzol (Thermo Fisher Scientific) for RNA isolation.

### Lipid labelling of vesicles

UPEC MVs containing 120μg of protein were labelled with 2μM of PKH67 (Thermo Fisher Scientific) in 2mL final volume by following manufacturers instructions. The labelled MV pellet was washed with RPMI and re-centrifuged at 75,000 *xg* for 1 hour to re-pellet the labelled MVs and to remove unincorporated dye.

5637 bladder carcinoma cells were seeded (20,000 cells/well/200μL) into Ibidi culture plates (Ibidi GmbH) in full growth media and incubated for 15hr at 37°C. 10μg of PKH67-labelled MVs were added to the cells and incubated for 2–15 hr at 37°C. Treated cells were stained with 10μM CellTracker™ Red CMTPX (Thermo Fisher Scientific), according to manufacturer’s instructions, fixed in 4% formaldehyde for 15 min and then permeabilised in 0.25% Triton for 20 min. Cells were further stained for 5 minutes with 600nM DAPI (Thermo Fisher Scientific), immersed in Citifluor AF3 anti-fading agent (Citifluor Ltd.) and imaged using confocal microscopy ZEISS LSM 710 Inverted Confocal Microscope.

### Labelling of bacterial RNA

To label MV RNA, UPEC 536 were grown with 100μM 5-ethynyl uridine (5EU; Thermo Fisher Scientific) for 6h at 37°C, shaking at 200rpm, prior to MV isolation. 5637 bladder carcinoma cells were treated with 5EU-labelled membrane vesicles (10μg protein) and stained with CellTracker Red as above. Cells were washed twice with 1% BSA in PBS, fixed and permeabilised as above before being incubated in Click-iT® reaction cocktail (Click-iT Cell Reaction Buffer Kit, Thermo Fisher Scientific) containing 2.5μM azide-containing Alexa Fluor 488 (Thermo Fisher Scientific), according to the manufacturer’s instructions. The nucleus was highlighted with DAPI and visualised as prior.

### Droplet digital RT-PCR

A standard curve was also generated by spiking different protein amounts of MV into 5637 cells lysed in trizol prior to RNA extraction. The spiking method ensured that the PCR product amplified was specific to the MV RNA and not due to background amplification of cellular transcripts.

Equal volumes of RNA from MV treatments of 5637 cells and the standards were used for cDNA synthesis using qScript cDNA supermix (Quanta Biosciences) following the manufacturers conditions. A ddPCR mix was prepared using cDNA diluted 1:5 for *csrC* with 100nM each F (5’- GAGGCGAAGACAGAGGATTG) and R (5’- TTTTTCCATTAGCCGGAACA) primers and ddPCR EvaGreen master mix (Bio-Rad) in a 22μL mix. Droplets were prepared by mixing 20μL PCR mix with 70μL EvaGreen generation oil in a QX100 droplet generator and then 40μL transferred to C1000 thermal cycler to cycle as per the manufacturers instructions (95°C 5min, followed by 40 cycles of 95°C 30s, 60°C 60s, then 4°C 5min and 90°C 5min). After PCR amplification, droplets were read using a QX200 Droplet Reader and analysed using QuantaSoft software.

## Results

### UPEC strain 536 releases RNA

If our hypothesis that RNA signals are released by UPEC is correct we would expect to find RNA in cell-free supernatants from bacterial culture. Spent supernatants were collected from UPEC 536 cultured overnight to stationary phase. Cultures contained approximately 2x10^9^ colony forming units/mL and 96.9±0.02% viable cells. Extracellular RNA was quantified at approximately 40ng/mL in these supernatants following passage through 0.22μm filters to remove intact bacteria.

### RNA is associated with UPEC membrane vesicles

**Fig 1:**
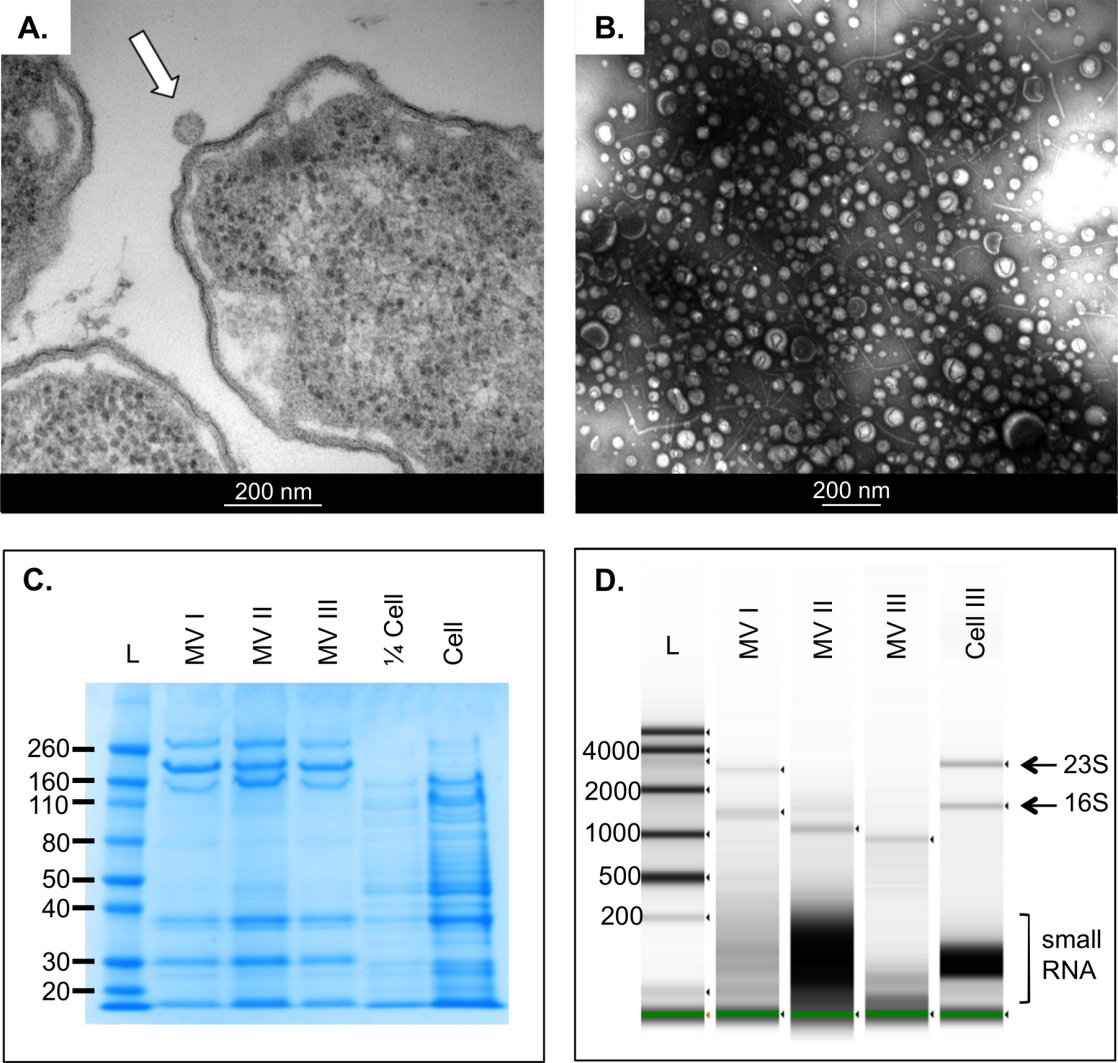
Bacteria release membrane vesicles that associate with protein and RNA. A. Contrast electron microscopy of budding UPEC with a white arrow pointing to the released MV. B. Contrast electron microscopy of isolated vesicle preparation by ultracentrifugation. C. Coomassie stained protein gel of MVs isolated from UPEC D. Agilent Tapestation gel for RNA from three replicate MVs isolated from UPEC plus one donor cell RNA. Intact ribosomal bands are labelled 23S and 16S as are the small RNA fragments. The green line marks the internal loading marker.

UPEC 536 were shown to release MVs during routine culture in iron replete RPMI medium as visualised by TEM (Fig 1A). These MVs could be isolated easily using simple filtration and ultracentrifugation and were validated using TEM (Fig 1B). Nanosight particle tracking analysis (NTA) was used to estimate the quantity of vesicles released by stationary phase UPEC at ~2x10^9^ MV per mL of culture. Protein banding patterns were also consistent between MV preparations and differ distinctly from donor bacterial cells (Fig 1C). We were unable to detect any DNA associated with the MVs but, consistent with our hypothesis, MVs were found to associate with RNA (Fig 1D) comprising both intact ribosomal RNAs and small RNAs of less than 200nt in size. The apparent variability in the RNA size patterns observed between replicate MV preparations we attribute to at least three factors: firstly the contributions from the underlying natural variation caused during bacterial culture. Secondly the likelihood of some unavoidable fragmentation of the isolated RNA during the lengthy MV processing, and thirdly due to slight variation in loading when handling small, dilute volumes, but which are detectable by the very high sensitivity of the Tapestation analysis. In comparison to the RNA yields from the crude filtered culture media, our isolated MV-RNA was calculated to comprise ~3% of all of the total extracellular RNA.

**Fig 2:**
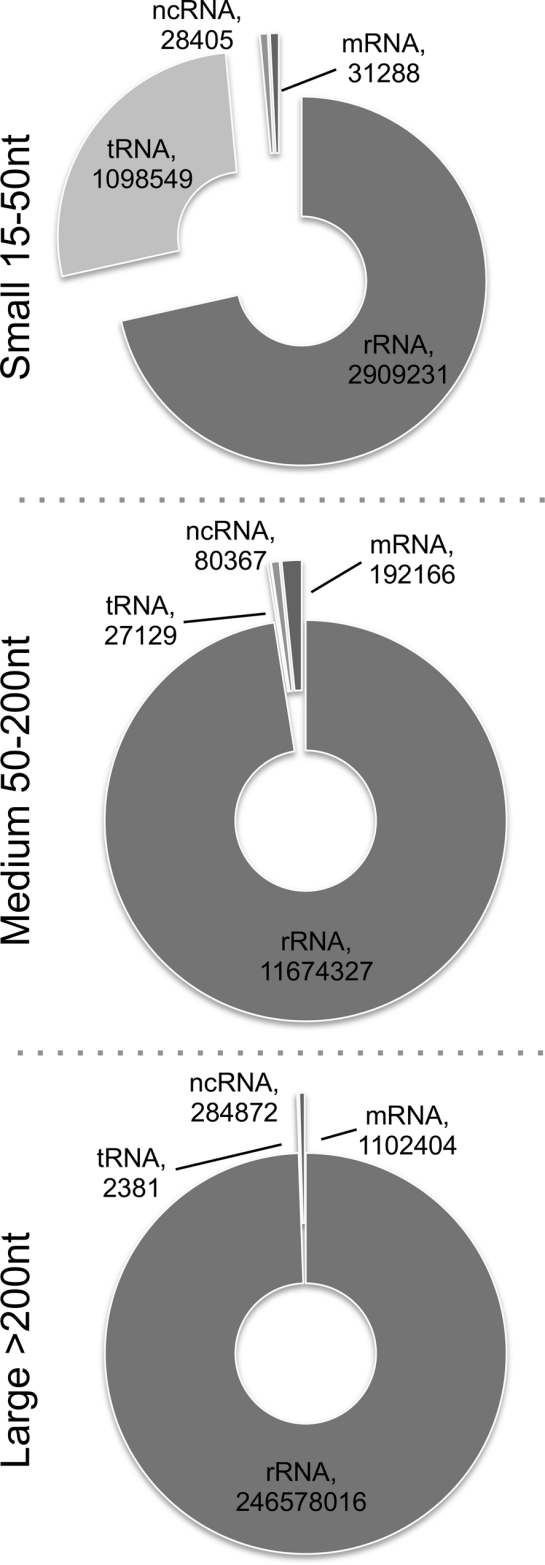
Populations of RNAs found in vesicles from three size separated libraries. RFAM and RefSeq annotated sequencing reads classified by RNA-type after alignment to UPEC 536 genome.

The RNA profiles of UPEC MVs were investigated using a new modified RNA-sequencing protocol. Three RNA libraries were prepared from each of two isolations of MVs from UPEC to comprise RNAs of 15-50nt, 50-200nt and 200+nt. This full size range fractionation allowed us to look at bacterial mRNA, ncRNAs and small RNAs within a single sample. When reads were matched to the *E*. *coli* 536 (O6:K15:H31) genome with annotation in SeqMonk for RFAM and RefSeq genes, it was found that each size library had a distinct content of RNAs (Fig 2, S1 Table). The MV RNA comprised reads from the vast majority of annotated genes in the UPEC 536 genome.

Due to the overall abundance of ribosomal RNA, this was as expected the most common read match in all size libraries. Interestingly, we found that the largest proportion of transfer RNA reads were found in the small library (27%), rather than the medium size (0.23%) in which we would have expected these mature ~90nt RNAs to reside. We also identified varying proportions of reads derived from ncRNA and mRNA genes in each of the sized libraries. The protein coding RNAs (e.g. hemolysin A and S-fimbrial-adhesin A) associated with MVs (S1 Table) were not enriched in function and were not preferentially derived from specific genomic locations such as pathogenicity islands in UPEC 536. Their presence in the large size libraries suggests that the full-length mRNAs are secreted in the MVs.

### RNA fractionates with MVs by DGC

**Fig 3:**
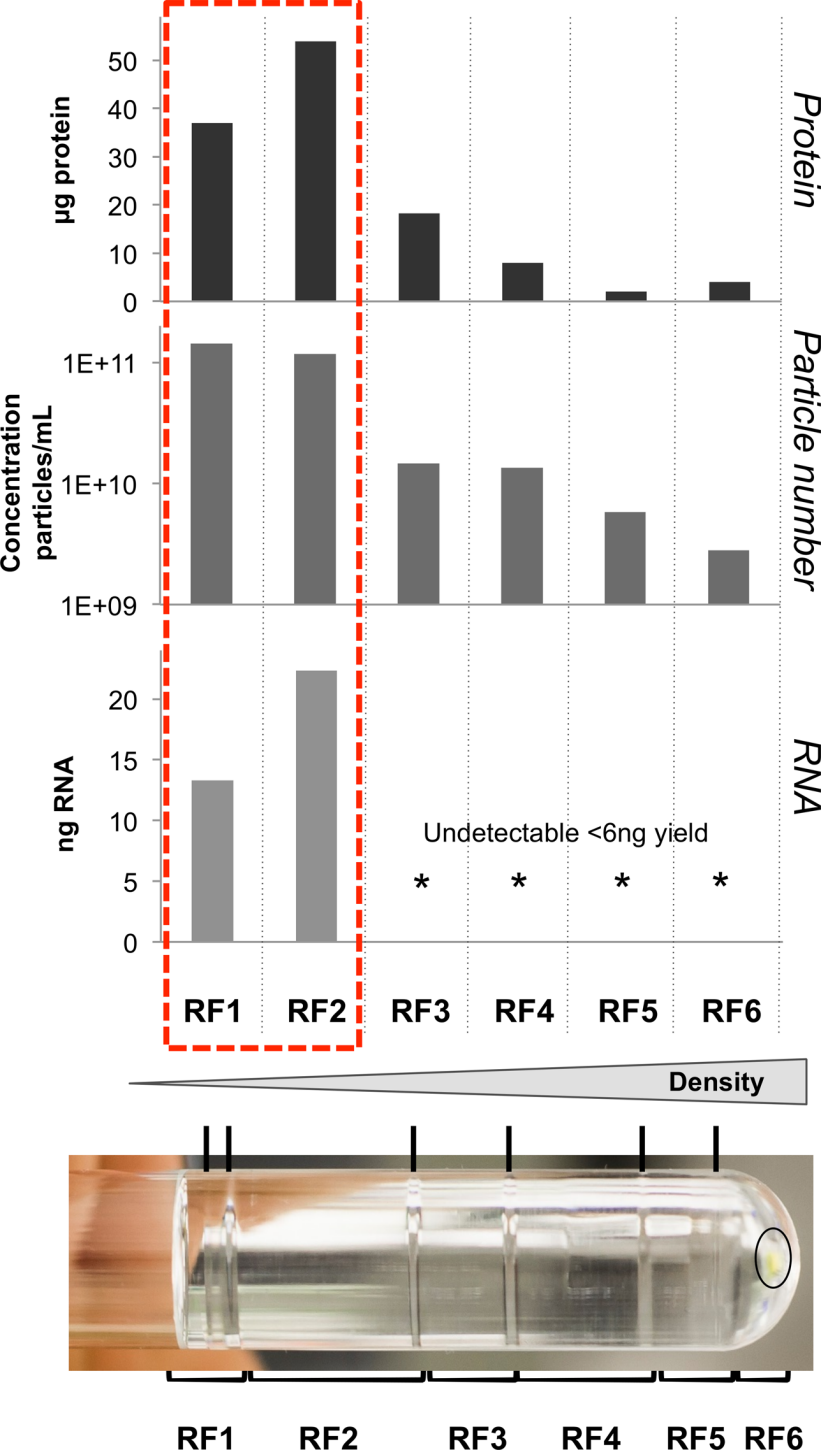
Density Gradient Centrifugal Purification confirms that the RNA is associated with the MV and protein dense fractions. At the bottom is the DGC banding pattern of UPEC MVs with bands noted by black lines and brackets highlighting the fractions (RF) taken. The density of the fractions increases down the gradient from RF1 to RF6. An oval shows the orange pellet that is seen when UPEC are grown with iron. Protein quantity (μg/fraction) in isolated fractions were determined by BCA. Isolated RNA quantity from each fraction presented in ng/fraction. NTA analysis of particle count per mL in each fraction. The dotted red box highlights the fractions that contain RNA and concomitantly contain the majority of the protein and particles.

The RNA we sequenced was isolated from an initial MV preparation using a simple filtration and ultracentrifugation protocol and may arguably have included RNAs associated with non-MV contaminants in the sample. To exclude this possibility we quantified the RNA after further DGC fractionation for MVs isolated from three independent cultures. Fig 3 illustrates the findings from a single representative preparation. All of the detectable RNA was consistently associated with particle and protein-rich fractions following DGC supporting the assumption that the RNA we originally sequenced was that primarily associated with MVs. A second MV preparation was assessed by TEM (S1 Fig) to confirm MV isolation and highlights the heterogeneity of the MV fractions. The same finding, of RNA being associated with MVs was also confirmed using a second purification technique, size exclusion chromatography (data not shown).

### MV cargo transport into epithelial cells

**Fig 4:**
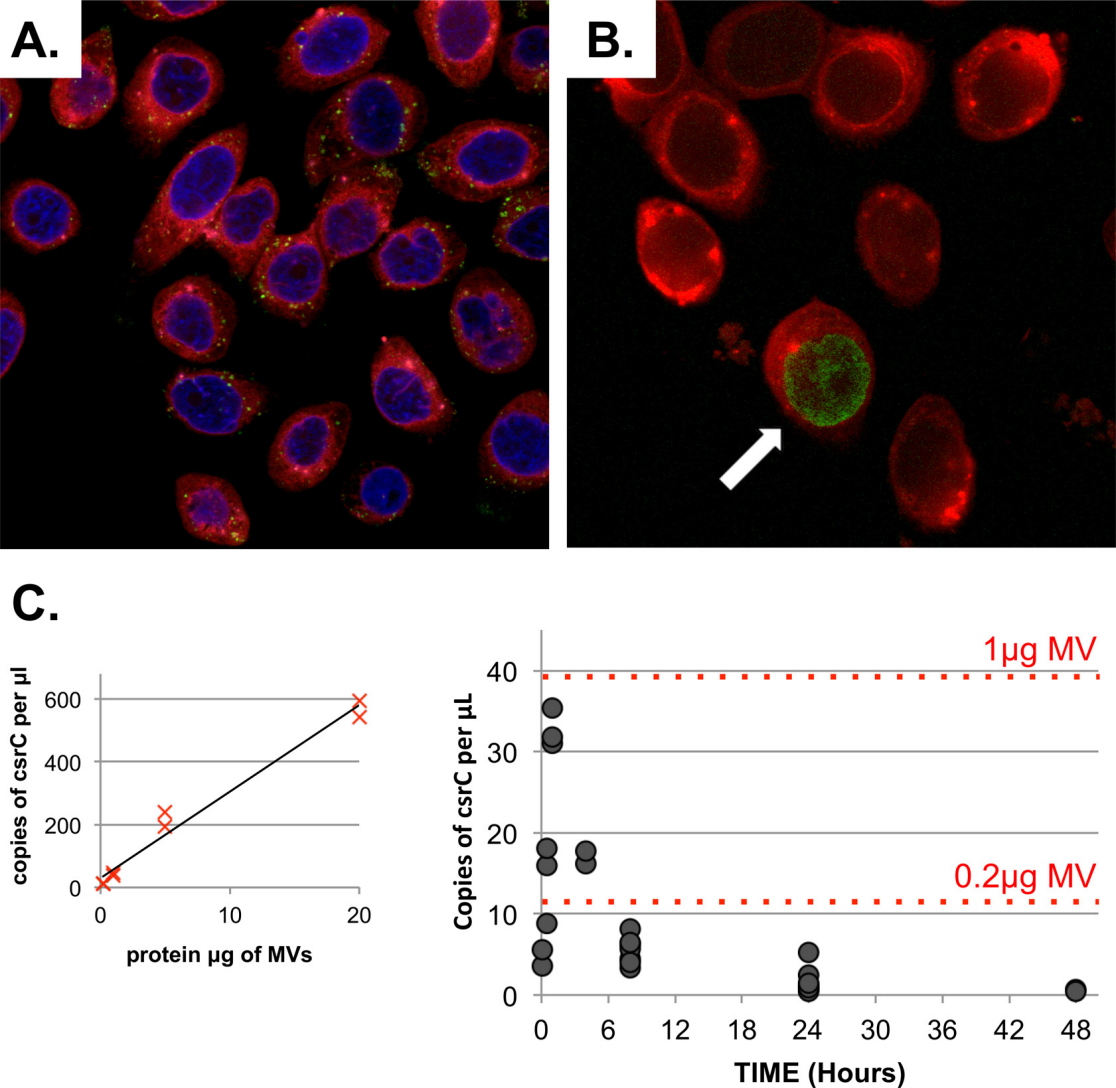
UPEC MV and their RNA cargo are delivered into human bladder cells *in vitro*. A. Confocal image of 5637 cells (red) stained with DAPI blue nuclear stain after treatment for 15 hr with 50μg/mL PKH26 green vesicles. B. Confocal image of 5637 cells (red) after treatment for 15 hr with 50μg/mL 5EU-labelled RNA vesicles (green with an arrow) C. Droplet digital RT-PCR validation of UPEC *csrC* rRNA into cells treated with 100μg/mL MVs across a 48 hr timeframe. Each treatment timepoint repeated in at least duplicate as represented by a closed spot. Red dotted lines mark the copies of *csrC* per μL from a standard curve of MV protein equivalents shown on the left.

The fact that UPEC MVs associate with specific RNAs led us to investigate whether it could be directly delivered to cells, to play a proposed role in cell-cell communication with respect to human infection. By applying lipid PKH67 stained MVs to cultured human bladder cells we confirmed that from 2 hr post-application stained vesicles could be detected within the cytoplasm of the treated cells (Fig 4A), often localising to a perinuclear position in the recipient cell. Over time this uptake continues, with for example, more labelled vesicles visible in the cells at 15 hr versus 2 hr.

We then looked at whether we could track uptake of MV RNA cargo into treated host cells by labelling UPEC nascent RNA in the bacteria before it was packaged with MVs. This was achieved by adding an excess of a modified uracil, 5EU (5-ethynyl uridine), which was incorporated into the newly synthesised and MV associated RNA. The level of 5EU incorporation and packaging was low because we used the least 5EU possible for nascent RNA labelling, but this was sufficient to show the uptake of MVs into a selection of bladder cells by 15–16 hrs. The cellular localisation was observed in multiple replicates to be both in the cytoplasm and intriguingly in the nucleus (Fig 4B). Due to the limitations of the labeling and the fact that only a small percentage of the MV-RNA is delivered we cannot further comment from this study on its most common and precise cellular localisation after uptake. This will require dedicated studies beyond this publication’s scope.

To further confirm uptake of bacterial RNA as an MV cargo, we performed a time course experiment and assayed the transfer of the bacterial *csrC* ncRNA into treated bladder cells by droplet digital PCR. This validation showed a rapid uptake of bacterial RNA into the cells, seen even at the first 5 minute timepoint, which peaked at 1 hr and then declined to almost undetectable by 48 hrs (Fig 4C). Based on a standard curve, when cells were treated with 100μg whole MVs (by protein content) the maximal uptake of their RNA cargo was equivalent to 0.8μg MV, so approximately 1% of the RNA is successfully transferred into cells.

## Discussion

In this study we report the first detailed and comprehensive characterisation of the bacterial RNA associated with the MVs produced by the Gram negative pathogen, *Escherichia coli* strain 536. This RNA cargo was sequenced and shown to comprise all forms of known RNA, including ribosomal, transfer, small RNA and mRNA. To support a role for this RNA in UPEC infection, we demonstrated that MVs and their associated RNA entered cultured bladder cells.

### MV associated RNA

The roles of bacterial MVs are under increasing scrutiny, particularly in terms of their protein and lipid cargo, and especially in infection scenarios. The potential of MVs to deliver a bioactive RNA to a target cell has been reported only five times, three of these in the past two years, highlighting the very recent but now growing interest in this phenomenon. In 1989, Dorward *et al*. reported an ‘RNA signature’ in MVs of *Neisseria gonorrhoeae* [10, 18]. Since then, Biller *et al*. (2014) assessed the mRNA content of vesicles released by *Prochlorococcus*, a marine picoplankton[10]. This was followed by two further studies in 2015, one again performed mRNA sequencing this time on MV-RNA derived from the pathogen *Vibrio cholerae* [12]. In the latter study the authors interestingly noted that whether the RNA in internal or external to the MVs was inconclusive when analysed after treatment with RNase. Indeed our own trials with RNAse digestion have found it to be inefficient for complete hydrolysis of smaller RNAs which may have RNase resistant secondary structures and/or be associated with proteins in Rnase stable complexes.

In the final paper to report MV-RNA, Ghosal *et al*. [11] performed small RNA-sequencing of the extracellular RNA derived from cultures of a non-pathogenic laboratory strain of *E*. *coli* grown in Luria-Bertani broth, a medium that does not claim to mimic the host environment [19], identifying specific packaging of small RNA into MVs. In contrast to our findings, their data reported that the vast majority of the MV-RNA was <200nt in size. Our own study, by comparison, has examined a human uropathogen, grown in physiological RPMI media, in which we undertook comprehensive sequencing of all the RNA content without selection bias. As a result we are able to form the first comprehensive picture of the RNA content of MVs from a human pathogen, beyond that destined for translation.

By splitting the RNA into three different ‘sized’ libraries we have assessed the complete profile of MV associated RNA from UPEC 536. Each library differed in its contents but all were dominated by abundant ribosomal RNA (71.5–99.4% annotated reads) which is not seen so commonly in eukaryotic vesicles [20]. Once the rRNA was excluded, the next most common reads mapped to tRNAs, predominantly in the small sized library (27%) showing that they are often fragmented from their mature sizes of 70-90nt. This fragmentation was specific to isoacceptor type and often dominant for either the 5’ or the 3’ end of the tRNA, a phenomenon reported in small RNA-sequencing libraries ranging from human biofluids [21] to Toxoplasma and Plasmodium parasites grown *in vitro* [22] and most recently confirmed in MV and extracellular RNA from *E*. *coli* K-12 [11]. Our MV RNA discovery could be very relevant, as Furuse *et al*. [23] have recently reported that tRNA fragments from *Chlamydia* and *Legionella* can be incorporated in the host RNA-induced silencing complex (RISC) and thus could be involved in direct subversion of host gene translation and mRNA stability.

After the rRNA and tRNA the next most common reads matched to protein coding mRNAs. These varied in abundance across the sized libraries (0.44–1.6%) but read coverage suggests that they are full-length mRNAs. Although present in our sequencing data we did not detect an *enrichment* specifically for RNAs transcribed from the genomic regions of each the five major UPEC 536 pathogenicity islands [24]. However RNA for a few individual genes contained within these pathogenicity islands including virulence factors hemolysin A (*hlyA*) and S-fimbrial-adhesin A (*sfaA*) [25, 26], were present in the MVs (S1 Table). The most common mRNAs found associated with MVs, alongside *ompA*, were from a ~42kb region encompassing ECP_1132 to ECP_1186, mostly prophage-derived genes. These prophage regions can interestingly be classed as further pathogenicity islands in UPEC [27]. Small RNA fragments were also detected derived from these phage genes. A recent article reports that enterohemorrhagic *E*. *coli* (EHEC) also makes detectable small RNAs from such phage genomic regions [28] with possible roles for these in regulation of gene expression to confer a growth advantage during host colonisation.

The final class of RNAs found in MVs were small non-coding RNAs. The annotation of this class of RNAs in the UPEC 536 genome is limited but includes stress responsive genes such as *oxyS*, *ryhB* and *csrC*. Based on their presence in specific ‘sized’ libraries and read coverage, these also appear to be packaged in their full-length forms. Cellular uptake of readily ‘active’ full-length RNA has been reported for mammalian mRNAs and regulatory RNAs transported in exosomes [29] but not for bacteria.

We also confirmed that the major proportion of RNA we isolated for our sequencing was co-fractionated with the MVs and protein using density gradient centrifugation and subsequent molecular analyses. Therefore the presence of ‘contaminating’ RNA in our simpler isolation method for the sequenced MVs, for example that associated with just protein aggregates was negligible. Thus the sequenced RNA can be considered as representative of that associated with the expected normal heterogenous population of MVs produced by UPEC.

### How does the RNA associate with the MVs?

There are several possibilities for RNA association with MVs. Firstly the RNA may be simply tightly attached to the outside of the MVs, associating in this way after secretion from the bacterium, and comprising extracellular RNA released by general bacterial cell lysis. Secondly this could be an active and selective mechanism of RNA incorporation into MVs; thirdly it may just represent RNA in the cytoplasm being non-specifically enveloped within vesicle blebs; finally it may be due to RNA being passengers on MV-bound proteins. All of these could be operational in bacterial culture. Prokaryotic RNA is non-randomly distributed with at least four patterns of mRNA localisation recognised [30]. Bacterial mRNAs are often found at the sites of their future protein products [30] thus it may be proposed that MVs would contain RNA for MV packaged proteins. Indeed many mRNAs for many membrane proteins were present in the MVs such as *ompA*, *lpp* and *tonB*. Furthermore, some of the proteins reported as packaged into *E*. *coli* MVs also bind RNA such as the protein chaperone GroEL, tRNA synthetases and ribosomal proteins [31]. EF-Tu is also commonly found in MV preparations [32], linked to immunogenicity [33, 34] and also binds tRNA, transfer messenger RNA (tmRNA) and ribosomes in its function as a translation elongation factor [35].

### MV RNA role in host infection?

The packaging of molecules into MVs allows protection from external degradation and specific delivery to target host cells. Indeed our own MV preparations carried a range of RNAs including long intact RNAs. These RNA components, if active, could confer a selective advantage to the bacteria, either through RNA exchange signalling between different bacteria, or similarly, directly between the bacterium and host. Focusing on the latter, we have shown that MVs are internalised into cultured human epithelial cells. Although technically limited by the amount of 5EU labelled RNA that could be packaged into MVs, visualisation of 5EU MV-RNA by confocal microscopy confirmed cargo internalisation in the host cell. Further ddPCR of the *csrC* RNA delivered by MVs in to cultured bladder cells confirmed that RNA is internalised, with approximately 1% of the cargo delivered into the cells. This relatively low rate of RNA delivery by MVs unfortunately limited our ability to perform a full time-course microscopy to localise and track the uptake of MV-RNA. However, the finding of labelled bacterial MV RNA in the cell nucleus after 16 hrs in some of the cells is intriguing, particularly with small RNAs now reported to be involved in epigenetic regulation by alteration of chromatin state through methylation of histones and DNA [36, 37].

Some of the proposed actions of packaged RNAs have been discussed above; with hijack of the RNA-inhibition system by tRNAs and miRNA-like RNAs a possibility for some of the MV associated content. These mechanisms are proposed to ultimately lead to a change in host gene expression that is beneficial to the pathogen and its survival.

## Conclusions

This study provides further important new support for the plausible role of bacterial MV-RNA as a novel bacteria-host signaling molecule and adds to the growing literature characterising prokaryotic MVs. We have confirmed here that UPEC 536 releases MVs that associate with RNA. This RNA comprises much of the bacterial transcriptome in a largely intact form. Furthermore we have found that the MVs are capable of delivering their associated RNA cargo into treated host cells which poses an interesting hypothesis of whether this RNA could act as a novel signaling molecule in uropathogenesis.

## Supporting Information

S1 Fig Transmission electron microscopy of density gradient fractions DGC fractions are labeled RF1 to RF6 and two photos are shown for each fraction, either two representations taken at a single magnification or at two different relevant magnifications. Scale bars are shown and the sizes of some identified vesicles are labeled.
(PDF)

S1 Table Full summary of annotated gene matches in RNA-seq data. Matched sequencing reads listed by annotated gene feature in each biological sample. Columns with reads (K-S) are split into libraries, small (<50nt), med (50-200nt), large (>200nt) by replicate and also presented as the sum of these (columns M, P, S).
(XLSX)
