# Supplementary figures and images for: Uropathogenic Escherichia coli Releases Extracellular Vesicles That Are Associated with RNA
Source: PLoS One. 2016 Aug 8;11(8):e0160440. doi: 10.1371/journal.pone.0160440 (PMC4976981; doi:10.1371/journal.pone.0160440)

TEM: UPEC RF MV preparation

Magnification 1

Magnification 2

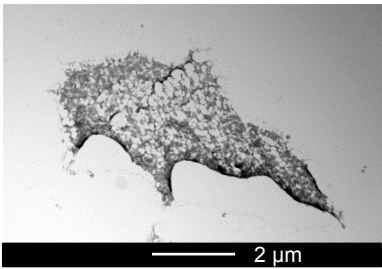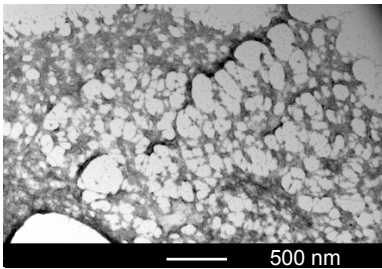

RF1

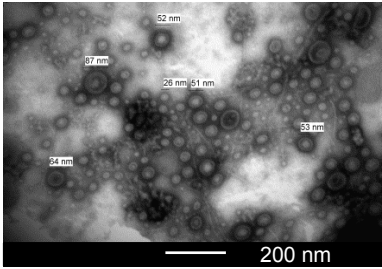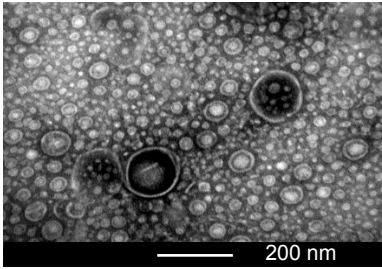

RF2

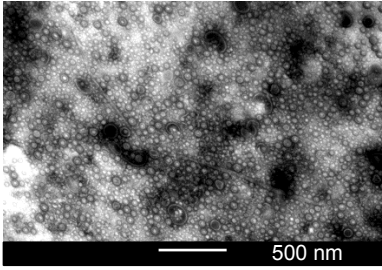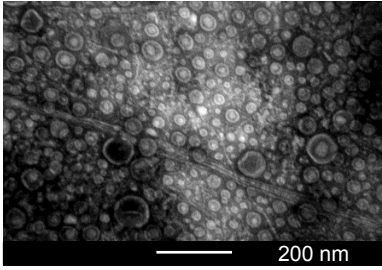

RF3

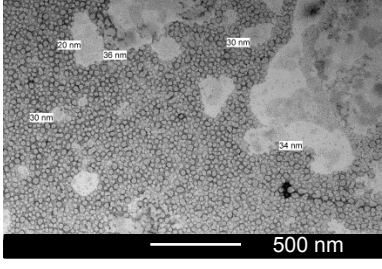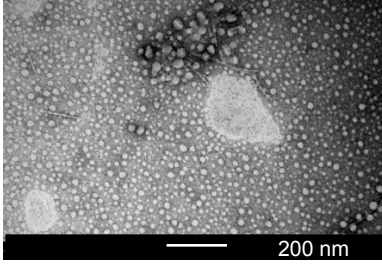

RF4

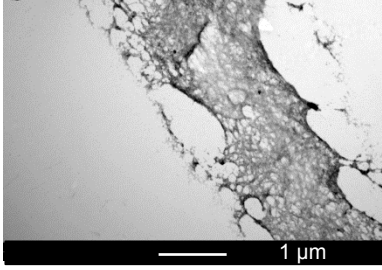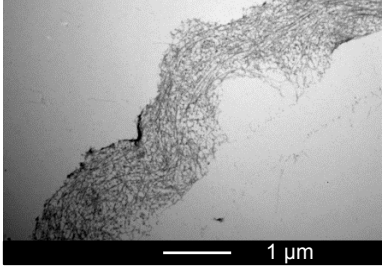

RF5

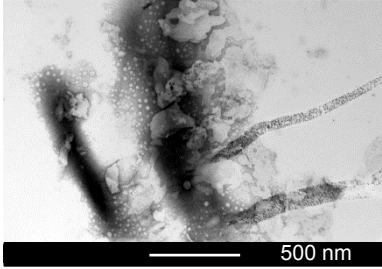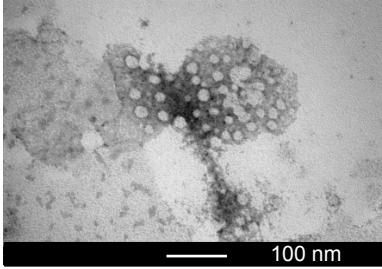

RF6

Supplement: S1 Fig — Scale bars are shown and the sizes of some identified vesicles are labeled. (PDF) [file pone.0160440.s001.pdf]
